# Identification of a novel SARS-CoV-2 P.1 sub-lineage in Brazil provides new insights about the mechanisms of emergence of variants of concern

**DOI:** 10.1093/ve/veab091

**Published:** 2021-12-15

**Authors:** Tiago Gräf, Gonzalo Bello, Taina Moreira Martins Venas, Elisa Cavalcante Pereira, Anna Carolina Dias Paixão, Luciana Reis Appolinario, Renata Serrano Lopes, Ana Carolina Da Fonseca Mendonça, Alice Sampaio Barreto da Rocha, Fernando Couto Motta, Tatiana Schäffer Gregianini, Richard Steiner Salvato, Sandra Bianchini Fernandes, Darcita Buerger Rovaris, Andrea Cony Cavalcanti, Anderson Brandão Leite, Irina Riediger, Maria do Carmo Debur, André Felipe Leal Bernardes, Rodrigo Ribeiro-Rodrigues, Beatriz Grinsztejn, Valdinete Alves do Nascimento, Victor Costa de Souza, Luciana Gonçalves, Cristiano Fernandes da Costa, Tirza Mattos, Filipe Zimmer Dezordi, Gabriel Luz Wallau, Felipe Gomes Naveca, Edson Delatorre, Marilda Mendonça Siqueira, Paola Cristina Resende

**Affiliations:** Plataforma de Vigilância Molecular, Instituto Gonçalo Moniz, Fiocruz, Salvador, Bahia 40296-710, Brazil; Laboratório de AIDS e Imunologia Molecular, Instituto Oswaldo Cruz, Fiocruz, Rio de Janeiro 21040-900, Brazil; Laboratório de Vírus Respiratórios e do Sarampo (LVRS), Instituto Oswaldo Cruz, Fiocruz, Rio de Janeiro 21040-900, Brazil; Laboratório de Vírus Respiratórios e do Sarampo (LVRS), Instituto Oswaldo Cruz, Fiocruz, Rio de Janeiro 21040-900, Brazil; Laboratório de Vírus Respiratórios e do Sarampo (LVRS), Instituto Oswaldo Cruz, Fiocruz, Rio de Janeiro 21040-900, Brazil; Laboratório de Vírus Respiratórios e do Sarampo (LVRS), Instituto Oswaldo Cruz, Fiocruz, Rio de Janeiro 21040-900, Brazil; Laboratório de Vírus Respiratórios e do Sarampo (LVRS), Instituto Oswaldo Cruz, Fiocruz, Rio de Janeiro 21040-900, Brazil; Laboratório de Vírus Respiratórios e do Sarampo (LVRS), Instituto Oswaldo Cruz, Fiocruz, Rio de Janeiro 21040-900, Brazil; Laboratório de Vírus Respiratórios e do Sarampo (LVRS), Instituto Oswaldo Cruz, Fiocruz, Rio de Janeiro 21040-900, Brazil; Laboratório de Vírus Respiratórios e do Sarampo (LVRS), Instituto Oswaldo Cruz, Fiocruz, Rio de Janeiro 21040-900, Brazil; Laboratório Central de Saúde Pública do Estado do Rio Grande do Sul (LACEN-RS), Porto Alegre 90610-000, Brazil; Laboratório Central de Saúde Pública do Estado do Rio Grande do Sul (LACEN-RS), Porto Alegre 90610-000, Brazil; Laboratório Central de Saúde Pública do Estado de Santa Catarina (LACEN-SC), Florianópolis 88010-001, Brazil; Laboratório Central de Saúde Pública do Estado de Santa Catarina (LACEN-SC), Florianópolis 88010-001, Brazil; Laboratório Central de Saúde Pública do Estado do Rio de Janeiro (LACEN-RJ), Rio de Janeiro 20231-000, Brazil; Laboratório Central de Saúde Pública do Estado de Alagoas (LACEN-AL), Maceió 57036-000, Brazil; Laboratório Central de Saúde Pública do Estado do Paraná (LACEN-PR), Curitiba 80045-150, Brazil; Laboratório Central de Saúde Pública do Estado do Paraná (LACEN-PR), Curitiba 80045-150, Brazil; Laboratório Central de Saúde Pública do Estado de Minas Gerais (LACEN-MG), Belo Horizonte 30510-010, Brazil; Laboratório Central de Saúde Pública do Estado do Espírito Santo (LACEN-ES), Vitória 29052-121, Brazil; Instituto Nacional de Infectologia (INI), Fiocruz, Rio de Janeiro 21040-900, Brazil; Laboratório de Ecologia de Doenças Transmissíveis na Amazônia (EDTA), Instituto Leônidas e Maria Deane, FIOCRUZ, Manaus, Amazonas 69027-070, Brazil; Laboratório de Ecologia de Doenças Transmissíveis na Amazônia (EDTA), Instituto Leônidas e Maria Deane, FIOCRUZ, Manaus, Amazonas 69027-070, Brazil; Laboratório de Ecologia de Doenças Transmissíveis na Amazônia (EDTA), Instituto Leônidas e Maria Deane, FIOCRUZ, Manaus, Amazonas 69027-070, Brazil; Fundação de Vigilância em Saúde do Amazonas, Manaus 69093-018, Brazil; Laboratório Central de Saúde Pública do Amazonas, Manaus 69020-040, Brazil; Departamento de Entomologia, Instituto Aggeu Magalhães, Fiocruz, Recife, Pernambuco 50670-420, Brazil; Núcleo de Bioinformática (NBI), Instituto Aggeu Magalhães Fiocruz, Recife, Pernambuco50670-420, Brazil; Departamento de Entomologia, Instituto Aggeu Magalhães, Fiocruz, Recife, Pernambuco 50670-420, Brazil; Núcleo de Bioinformática (NBI), Instituto Aggeu Magalhães Fiocruz, Recife, Pernambuco50670-420, Brazil; Laboratório de Ecologia de Doenças Transmissíveis na Amazônia (EDTA), Instituto Leônidas e Maria Deane, FIOCRUZ, Manaus, Amazonas 69027-070, Brazil; Departamento de Biologia, Centro de Ciências Exatas, Naturais e da Saúde, Universidade Federal do Espírito Santo, Alegre 29500-000, Brazil; Laboratório de Vírus Respiratórios e do Sarampo (LVRS), Instituto Oswaldo Cruz, Fiocruz, Rio de Janeiro 21040-900, Brazil; Laboratório de Vírus Respiratórios e do Sarampo (LVRS), Instituto Oswaldo Cruz, Fiocruz, Rio de Janeiro 21040-900, Brazil

**Keywords:** SARS-CoV-2, genomic surveillance, Brazil, variant of concern Gamma, lineage P.1

## Abstract

One of the most remarkable severe acute respiratory syndrome coronavirus 2 (SARS-CoV-2) variants of concern (VOC) features is the significant number of mutations they acquired. However, the specific factors that drove the emergence of such variants since the second half of 2020 are not fully resolved. In this study, we describe a new SARS-CoV-2 P.1 sub-lineage circulating in Brazil, denoted here as Gamma-like-II, that as well as the previously described lineage Gamma-like-I shares several lineage-defining mutations with the VOC Gamma. Reconstructions of ancestor sequences support that most lineage-defining mutations of the Spike (S) protein, including those at the receptor-binding domain (RBD), accumulated at the first P.1 ancestor. In contrast, mutations outside the S protein were mostly fixed at subsequent steps. Our evolutionary analyses estimate that P.1-ancestral strains carrying RBD mutations of concern probably circulated cryptically in the Amazonas for several months before the emergence of the VOC Gamma. Unlike the VOC Gamma, the other P.1 sub-lineages displayed a much more restricted dissemination and accounted for a low fraction (<2 per cent) of SARS-CoV-2 infections in Brazil in 2021. The stepwise diversification of lineage P.1 through multiple inter-host transmissions is consistent with the hypothesis that partial immunity acquired from natural SARS-CoV-2 infections in heavily affected regions might have been a major driving force behind the natural selection of some VOCs. The lag time between the emergence of the P.1 ancestor and the expansion of the VOC Gamma and the divergent epidemic trajectories of P.1 sub-lineages support a complex interplay between the emergence of mutations of concern and viral spread in Brazil.

## 1. Introduction

The emergence of the severe acute respiratory syndrome coronavirus 2 (SARS-CoV-2) variant of concern (VOC) Gamma (lineage P.1) in the Brazilian Amazonas state around November 2020 (Faria et al. 2021; Naveca et al. 2021) and its rapid dissemination to other regions were associated with a significant COVID-19 epidemic wave that collapsed the Brazilian health system during early 2021. The VOC Gamma, as with the other described VOCs Alpha (lineage B.1.1.7), Beta (lineage B.1.351), and Delta (lineage B.1.617.2), harbors a larger number of lineage-defining mutations than other contemporaneous non-VOC. The VOC Gamma carry ten non-synonymous substitutions in the Spike (S) protein (L18F, T20N, P26S, D138Y, R190S, K417T, E484K, N501Y, H655Y, and T1027I), five non-synonymous mutations distributed in the NSP3 (S370L and K977Q), NSP13 (E341D), NS8 (E92K), and N (P80R) proteins, one deletion in the NSP6 (S106del, G107del, and F108del), and a four-nucleotide insertion at the ORF8/N intergenic region (ins28263) (Faria et al. 2021; Fujino et al. 2021).

The most commonly discussed hypothesis to explain the origin of the VOC Alpha is that this variant may have resulted from viral adaptation during a persistent individual infection (Rambaut et al. 2020), as those observed in patients with immunosuppression (Corey et al. 2021). This hypothesis, however, does not explain the viral diversity of lineage P.1 in the Amazonas. A previous study conducted by our group described a P.1 sub-lineage, here denoted as Gamma-like-I, that branched as a sister monophyletic clade with respect to Gamma and accumulated an unusually high number of genetic changes, including several Gamma-defining mutations in the S (L18F, P26S, D138Y, K417T, E484K, N501Y), NSP3 (K977Q), and N (P80R) proteins and some unique mutations in the NSP2 (K456R), NSP3 (T1189I), NSP6 (V149A), NSP13 (S74L), S (ins214 and D1139H), and NS8 (K2stop) proteins (Naveca et al. 2021). Such a finding supports that the Gamma-defining mutations did not accumulate in a unique long-term individual infection but were acquired at sequential steps during the evolution of lineage B.1.1.28 in Amazonas.

In this study, we describe a second P.1 sub-lineage that also branched as a sister monophyletic clade with respect to Gamma and harbors 15 Gamma lineage-defining mutations and six unique mutations. The description of this new P.1 sub-lineage allowed us to trace with more precision the evolutionary steps that resulted in the emergence of the VOC Gamma in Brazil. Our analyses also revealed that despite sharing crucial mutations in the RBD of the S protein and having arisen around the same time, the different P.1 sub-lineages displayed divergent patterns of epidemic spread in Brazil.

## 2. Materials and methods

### 2.1 Ethics statement

This study was approved by the FIOCRUZ-IOC (68118417.6.0000.5248 and CAAE 32333120.4.0000.5190) and the Amazonas State University Ethics Committee (CAAE: 25430719.6.0000.5016) and the Brazilian Ministry of the Environment (MMA) A1767C3.

### 2.2 SARS-CoV-2 whole-genome sequencing

A total of 4,421 SARS-CoV-2 positive samples diagnosed in Brazil between 1 August 2020 and 31 March 2021 were sequenced by the Fiocruz COVID-19 Genomic Surveillance Network. The SARS-CoV-2 whole-genomes (>99 per cent coverage) were recovered using Illumina sequencing protocols as previously described (Nascimento et al. 2020; Resende et al. 2020). The FASTQ reads obtained were imported into the CLC Genomics Workbench version 20.0.4 (Qiagen A/S, Denmark), trimmed, and mapped against the reference sequence EPI_ISL_402124 available in EpiCoV database in the GISAID (https://www.gisaid.org/). The alignment was refined using the InDels and Structural Variants module.

### 2.3 Maximum likelihood phylogenetic analyses

SARS-CoV-2 P.1 sequences here obtained were aligned with high quality (<5 per cent of N) and complete (>29 kb) B.1.1.28 sequences from Amazonas and P.1 sequences from Brazil that were available in the EpiCoV database in the GISAID (https://www.gisaid.org/) on 30 April 2021. This dataset (*n* = 627) was then aligned using MAFFT v7.475 (Katoh and Standley 2013) and subjected to maximum likelihood (ML) phylogenetic analysis using IQ-TREE v2.1.2 (Minh et al. 2020) under the GTR + F + R4 nucleotide substitution model, as selected by the ModelFinder application (Kalyaanamoorthy et al. 2017). Branch support was assessed by the approximate likelihood-ratio test based on the Shimodaira–Hasegawa procedure (SH-aLRT) with 1000 replicates. The sequence of ancestral nodes was reconstructed using Time-tree (Kumar et al. 2017) and their mutational profile was investigated using the Nextclade tool (https://clades.nextstrain.org). The temporal signal was assessed by the regression analysis of the root-to-tip genetic distance estimated from the ML tree against sampling dates using the program TempEst (Rambaut et al. 2016).

### 2.4 Bayesian phylogeographic analyses

A time-scaled phylogenetic tree of the B.1.1.28 Amazonian diversity plus lineage P.1 sequences was reconstructed using the Bayesian Markov Chain Monte Carlo (MCMC) approach implemented in BEAST 1.10.4 (Suchard et al. 2018). To reduce the computation time while preserving the early diversity of VOC Gamma, five genomes sampled per week in Amazonas during December 2020 were randomly chosen using Augur (Hadfield et al. 2018), totalizing a dataset of 175 genomes. A posterior distribution of trees was obtained using the GTR + F + G4 nucleotide substitution model, the exponential growth coalescent model (Drummond et al. 2005), and different molecular clock models (strict, uncorrelated relaxed, random local, and fixed local). For the fixed local clock (FLC) we allowed rates to change in the stem branch leading to each P.1 sub-lineages (Gamma and Gamma-likes) and the branch where all P.1 diversity coalesces. A relaxed uniform prior on substitution rates (7–70 × 10^–4^ subs/site/year) was used in all clocks, covering the mean earlier estimates for overall (Duchene et al. 2020; Ghafari et al. 2021) and VOC-stem (Tay et al. 2021) branches. The log marginal likelihood to compare molecular clock models was estimated using the generalized steppingstone sampling method (Baele, Lemey, and Suchard 2015). Ancestral sampling locations were inferred using a reversible discrete phylogeographic model (Lemey et al. 2009) where transitions between Brazilian states were estimated in a continuous-time Markov chain rate reference prior (Ma and Suchard 2008). MCMC was run for 50 × 10^6^ steps to ensure that the effective sample size of all parameters, after discarding 10 per cent of the chain as burn-in, was >200 as assessed in TRACER v1.7 (Rambaut et al. 2018). MCMC analyses were performed in duplicate to verify the convergence of independent chains. The maximum clade credibility (MCC) tree was summarized with TreeAnnotator v1.10.4. ML and MCC trees were visualized using FigTree v1.4.4 (http://tree.bio.ed.ac.uk/software/figtree/).

## 3. Results

**Figure 1.**
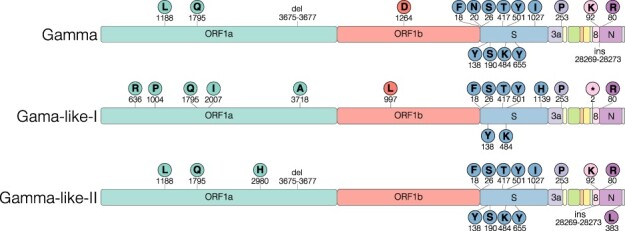
Characteristic mutations of Gamma and Gamma-related lineages. Schematic representation of the genomic organization of SARS-CoV-2 showing the open-reading frames and structura, and accessory proteins. The names of the genomic regions were indicated only where lineage-defining mutations (circles with one-letter amino acid code and the mutation position) were found.

The mutation profile analysis of 4,421 SARS-CoV-2 positive samples detected at different Brazilian states between 1 August 2020 and 31 March 2021 (Supplementary Fig. S1) revealed 61 lineage P.1 sequences (Supplementary Table S1) that harbor 15 out of 22 Gamma defining mutations, including: (1) the three mutations of concern at the receptor-binding domain (RBD) of the S protein (K417T, E484K, and N501Y), (2) four mutations in the amino(N)-terminal domain (NTD) of the S protein (L18F, P26S, D138Y and R190S), (3) one mutation close to the S1/S2 furin cleavage site (H655Y), (4) the deletion in the NSP6 (S106del, G107del, and F108del), and (5) the four-nucleotide insertion at ORF8/N intergenic region (ins28263) (Fig. 1). These new P.1 sequences, here denoted as Gamma-like-II, lack some of the Gamma-defining mutations at ORF1ab, NSP13, S, and NS8 and further displayed six unique substitutions at ORF1ab (D2980H, C8905T, C16954T, and G20931A), E/M intergenic region (A26492T), and N (P383L). The Gamma-like-II sequences also share nine lineage-defining mutations with the previously characterized Gamma-like-I clade (Fig. 1).

**Figure 2.**
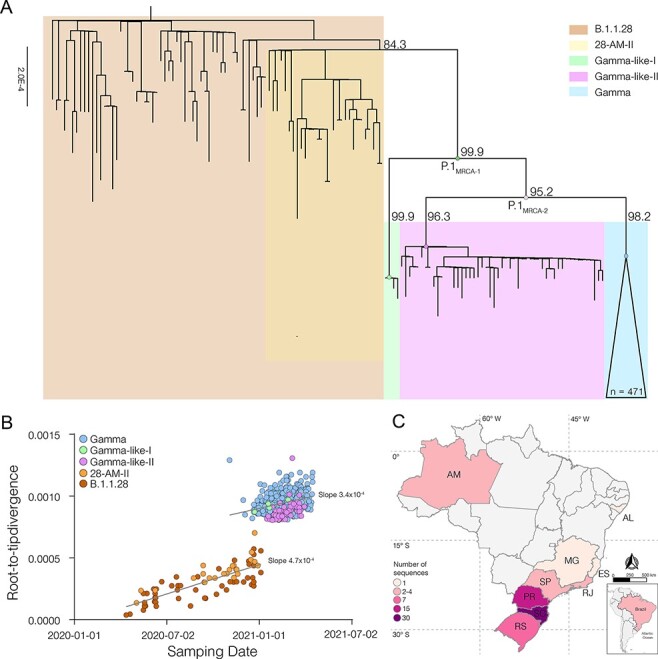
Genetic diversity and distribution of the B.1.1.28, Gamma, and Gamma-like lineages in Brazil. (A) ML phylogenetic tree of the B.1.1.28, Gamma, and Gamma-like lineages identified in Brazil. Each lineage was highlighted with colored boxes as indicated in the legend. The SH-aLRT support values are indicated in key branches, and branch lengths are drawn to scale with the lateral bar indicating nucleotide substitutions per site. Nodes representing the MRCA of each lineage and the MRCA of all Gamma and Gamma-related viruses (P.1_MRCA1_), and the MRCA of Gamma and Gamma-like-II (P.1_MRCA2_) are highlighted with circles. (B) Correlation between the sampling date of B.1.1.28, Gamma, Gamma-like-I, and Gamma-like-II and their genetic distance from the ML phylogenetic tree’s root. Each lineage was colored following the legend. The slope of each regression is indicated. (C) Geographic distribution and frequency of the Gamma-like-II lineage identified in Brazil. Brazilian states’ names follow the ISO 3166-2 standard. Color’s gradient represents the number of sequences identified in this study.

The ML phylogenetic analysis of B.1.1.28 Amazonian sequences (*n* = 83) and P.1 (*n* = 544) Brazilian sequences recovered in our study and those deposited in the EpiCoV database by 30 April 2021 showed that all Gamma-like-II sequences here described branched in a highly supported (SH-aLRT = 96 per cent) monophyletic clade (Fig. 2A). Clades Gamma-like-I and Gamma-like-II are not nested within the VOC Gamma but branch as sister monophyletic clades that evolved from a common ancestor and are classified as lineage P.1 according to the PANGO rules (https://github.com/cov-lineages/pango-designation/issues/77). Clades Gamma, Gamma-like-I, and Gamma-like-II were thus considered as different P.1 sub-lineages. Analysis of the temporal structure revealed that clades Gamma, Gamma-like-I, and Gamma-like-II accumulated a higher number of mutations when compared to B.1.1.28 contemporaneous sequences, but the divergence rate within lineage P.1 was similar to the ancestral lineage B.1.1.28, supporting that after an episodic acceleration of the molecular clock rate, both lineages evolved at roughly the same rate (Fig. 2B).

The clade Gamma-like-II was detected at nine different Brazilian states, mainly from the South and Southeast regions (Fig. 2C). The oldest sequence was detected in the Santa Catarina state on 16 January 2021 and the most recent one was identified in the Rio Grande do Sul state on 31 March 2021. Clade Gamma-like-II comprises a low fraction (2 per cent) of all SARS-CoV-2 Brazilian sequences genotyped between 1 January and 31 March 2021 but attained a relative high prevalence in the Southern states of Santa Catarina (10 per cent), Parana (10 per cent) and Rio Grande do Sul (5 per cent) (Table 1). Clade Gamma-like-I was restricted to a few sequences detected in the Amazonas (*n* = 3), Parana (*n* = 1), and Santa Catarina (*n* = 1) states between 23 December 2020 and 27 February 2021 (Table 1). The remarkable detection of both Gamma-like clades in Parana and Santa Catarina supports that P.1 sub-lineages that circulated at a very low prevalence in the Amazonas were occasionally disseminated to those Southern Brazilian states. The VOC Gamma, however, outcompeted both Gamma-like clades and became the most prevalent variant in Amazonas and all other Brazilian states during 2021 (Table 1).

**Table 1.** Prevalence of SARS-CoV-2 Gamma-like-I, and Gamma-like-II genomes per Brazilian State with the collection date from 1 January to 31 March 2021.

Country	Region	State	Lineage	Number of genomes^a^	Prevalence (%)
Brazil			Other	1231	36.2
			Gamma	2102	61.8
			Gamma-like-I	3	0.1
			Gamma-like-II	67	2.0
	North	Amazonas	Other	14	3.3
			Gamma	405	96.0
			Gamma-like-I	1	0.2
			Gamma-like-II	2	0.5
	Northeast	Alagoas	Other	68	51.5
			Gamma	63	47.7
			Gamma-like-II	1	0.8
	Southeast	Espírito Santo	Other	73	78.5
			Gamma	19	20.4
			Gamma-like-II	1	1.1
		Minas Gerais	Other	187	61.7
			Gamma	113	37.3
			Gamma-like-II	3	1.0
		Rio de Janeiro	Other	175	35.9
			Gamma	309	63.3
			Gamma-like-II	4	0.8
		Sao Paulo	Other	508	37.1
			Gamma	856	62.6
			Gamma-like-II	4	0.3
	South	Parana	Other	40	26.8
			Gamma	93	62.4
			Gamma-like-I	1	0.7
			Gamma-like-II	15	10.1
		Rio Grande do Sul	Other	64	46.4
			Gamma	67	48.6
			Gamma-like-II	7	5.1
		Santa Catarina	Other	102	32.9
			Gamma	177	57.1
			Gamma-like-I	1	0.3
			Gamma-like-II	30	9.7

a Genomes available at GISAID up to 31 April 2021.

**Figure 3.**
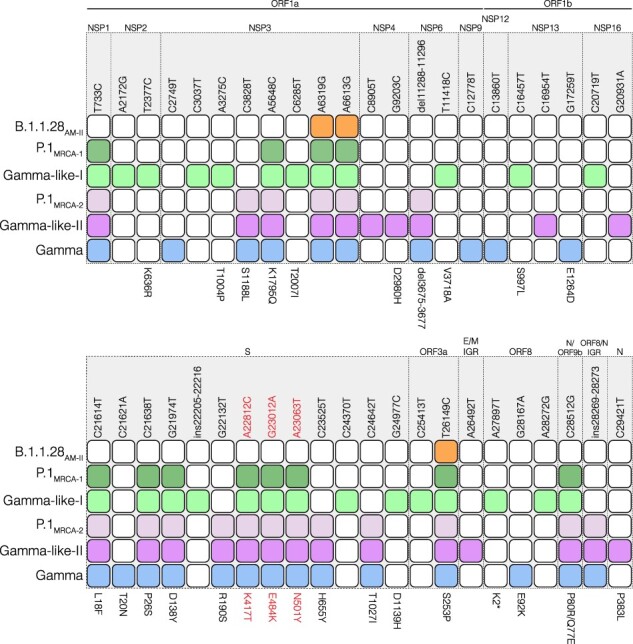
Evolutionary steps associated with the emergence of Gamma and Gamma-related lineages. Colored squares represent the node where the mutation emerged and was fixed during the diversification of the B.1.1.28 lineage in Brazil originating the Gamma, Gamma-like-I, and Gamma-like-II lineages. Nodes’ colors and topology are described in Fig. 2A. The genomic position of the polymorphism is indicated at the top and the amino acid change at the bottom. Mutations of concern are in red. IGR: Intergenic region.

The reconstruction of sequences at ancestral nodes provides a clear picture of the evolutionary steps that resulted in the different P.1 variants (Fig. 3). Three mutations were fixed in the basal B.1.1.28 Amazonian clade (previously named 28-AM-II) (Naveca et al. 2021) from which all P.1 sub-lineages evolved. Nine mutations were fixed in the following evolutionary step that gave origin to the most recent common ancestor (MRCA) of lineage P.1 (designated as P.1_MRCA1_). Six additional mutations were fixed in the evolutionary branch that gave origin to the MRCA of clades Gamma and Gamma-like-II (designated as P.1_MRCA2_), and additional 6–12 mutations were fixed in the branches that originate the MRCA of each clade. Six out of the nine (67 per cent) mutations in P.1_MRCA1_ were in the S protein (including the three mutations of concern in the RBD), while only seven out of 32 (22 per cent) mutations fixed in the subsequent steps were located in the S gene. It is also interesting to note that the total number of lineage-defining mutations accumulated by clades Gamma (*n* = 12), Gamma-like-I (*n* = 14), and Gamma-like-II (*n* = 12) since their divergence from ancestor P.1_MRCA1_ was almost the same.

To better resolve the evolution of lineage P.1 before the emergence of VOC Gamma in Amazonas and the spatio-temporal dissemination pattern of the clade Gamma-like-II, we conducted a Bayesian phylogeographic analysis of a dataset that combined all B.1.1.28 basal sequences from Amazonas, a subset of VOC Gamma viruses sampled in Amazonas in December 2020 and all Gamma-like sequences. We compared four different molecular clock models, including a FLC-stem model that allows the evolutionary rate to vary along the most internal branches of lineage P.1, before diversification of Gamma and Gamma-like clades. We found that the RLC ranked well below the other models (log Bayes Factor > 100) (Kass and Raftery 1995), the FLC-stem model had very similar performance compared with both strict and relaxed models (log Bayes Factor < 2), while the strict model performed somewhat worse than the relaxed one (log Bayes Factor = 3.1) (Supplementary Table S2).

**Figure 4.**
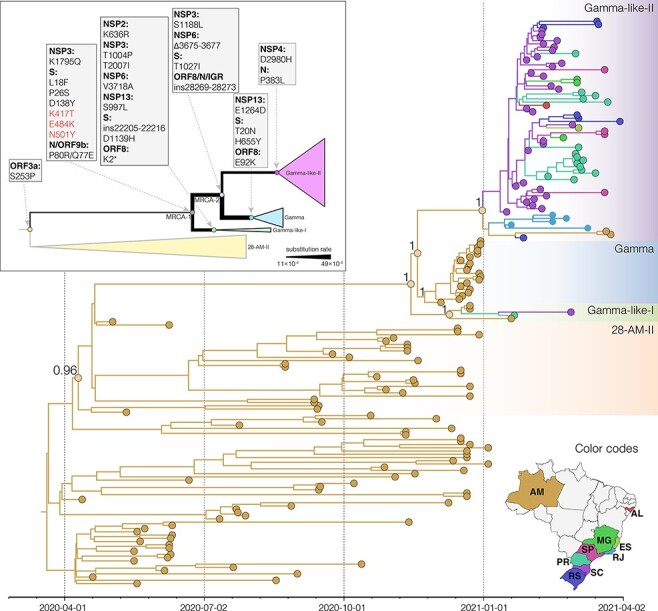
Bayesian phylogeographic analysis of the B.1.1.28, Gamma, and Gamma-related lineages. Tips and branches’ colors indicate the Brazilian state (ISO 3166-2 standard) of sampling and the most probable inferred location of their descendant nodes, respectively, as indicated in the map at the bottom. Branch posterior probabilities are indicated in key nodes. Boxes with different colors highlight the 28-AM-II, Gamma, Gamma-like-I, and Gamma-like-II lineages. All horizontal branch lengths are time-scaled, and the tree was automatically rooted under the assumption of the FLC-stem model. The inset shows a schematic tree representing the Gamma and Gamma-like diversification. Key nodes representing the MRCA of each lineage and the MRCA of all Gamma and Gamma-related viruses (labeled as MRCA1) and the MRCA of Gamma and Gamma-like-II (labeled as MRCA2) are highlighted with circles. The lineage-defining amino acid changes that differentiate each lineage are described in the gray boxes. The branch weights represent the rate of evolution estimated for each stem branch.

The background evolutionary rate of the FLC-stem model (7.2 × 10^–4^ subs/site/year) was equal to that estimated by the strict and relaxed clock models (Table 2) but suggested a much higher median substitution rate at the P.1_MRCA2_, Gamma and Gamma-like-I and -II stem branches (24.3–49.1 × 10^–4^ subs/site/year, respectively) and a slightly higher median rate at the P.1_MRCA1_ stem branch (11.2. × 10^–4^ subs/site/year) (Fig. 4). These distinct internal branch rates estimated by the FLC-stem model resulted in the recalibration of the node height of P.1_MRCA1_ and P.1_MRCA2_ ancestors, whose origin was estimated to be more recent under the FLC-stem model compared with other molecular clock models. Most notably, the P.1_MRCA1_ was estimated to have emerged in mid-October 2020 under the FLC-stem model, while the strict and relaxed clocks have estimated this ancestor to mid-August and early September 2020, respectively (Table 2). In contrast, the node height of Gamma and Gamma-like clades was very similar across the different molecular clock models that traced the origin of those P.1 sub-lineages between mid-November and late December 2020 (Table 2).

**Table 2.** Bayesian estimates of the time of P.1 and P.1-related most recent common ancestors using two different molecular clock models.

Ancestor	tMRCA (95% HPD)		
	Strict clock	Relaxed UCLD	FLC-stem
Background rate × 10^−4^ (95% HPD)	7.2 (7.0–7.8)	7.7 (7.0–9.0)	7.2 (7.0–7.7)
P.1_MRCA1_	15 August 2020 (06 July—21 September)	6 September 2020 (17 July—19 October)	21 October 2020 (18 September—13 November)
P.1_MRCA2_	29 September 2020 (29 August—28 October)	14 October 2020 (07 September—11 November)	5 November 2020 (16 October—21 November)
Gamma	16 November 2020 (02 November—29 November)	17 November 2020 (30 October—29 November)	20 November 2020 (06 November—30 November)
Gamma-like-I	14 December 2020 (30 November—22 December)	12 December 2020 (22 November—22 December)	11 December 2020 (23 November—22 December)
Gamma-like-II	9 December 2020 (17 November—25 December)	9 December 2020 (15 November—26 December)	5 December 2020 (14 November—22 December)

The phylogeographic reconstruction supports that, as expected, most ancestors during the diversification of lineage P.1 were probably located in the state of Amazonas (*Posterior State Probability* [*PSP*] = 1), with exception of the Gamma-like-II ancestor whose posterior probability was divided between Amazonas (*PSP* = 0.54) and Santa Catarina (*PSP* = 0.25) (Fig. 4). This analysis estimated that Santa Catarina was the most critical hub of dissemination of sub-lineage Gamma-like-II to other Brazilian states and further supports the establishment of local transmission networks of this sub-lineage in the states of Parana, Rio Grande do Sul and Rio de Janeiro. We estimated that the clade Gamma-like-II probably started to circulate in Santa Catarina on 8 January 2021 (95 per cent HPD: 29 December 2020–16 January 2021), in Rio de Janeiro on 14 January 2021 (95 per cent HPD: 05 January–19 January 2021), in Parana on 29 January 2021 (95 per cent HPD: 22 January–6 February 2021), and in Rio Grande do Sul on 3 February 2021 (95 per cent HPD: 25 January–9 February 2021).

## 4. Discussion

Our genomic surveillance identified a new P.1 sub-lineage, here denoted as clade Gamma-like-II, that shares a common ancestor and several lineage-defining mutations with the VOC Gamma and the clade Gamma-like-I, previously identified by our group (Naveca et al. 2021). The VOC Gamma and the two Gamma-like sub-lineages share a total of nine lineage-defining mutations, including many located in the RBD (K417T, E484K, and N501Y) and NTD (L18F, P26S, and D138Y) regions of the S protein.

The new clade Gamma-like-II comprises a low proportion (2 per cent) of all SARS-CoV-2 sequences in Brazil during the first months of 2021 but reached a relatively high prevalence (∼10 per cent) in the country’s South region. While the early diversification of lineage P.1 and the origin of clades Gamma and Gamma-like-I occurred in the Amazonas state, the origin of clade Gamma-like-II was traced to Amazonas or Santa Catarina states with a relatively high probability. Santa Catarina was pointed out as the most important hub of dissemination of clade Gamma-like-II to other Brazilian states. The great uncertainty in the location of the Gamma-like-II ancestor may reflect the low number of sequences from this clade detected in the Amazonas so far. Alternatively, a P.1 ancestral virus might have been introduced from Amazonas to Santa Catarina where it further spread, originating the clade Gamma-like-II that was subsequently re-introduced into the Amazonas state. The overall low fraction of SARS-CoV-2 positive samples sequenced in Brazil during early 2021 is an important limitation and may have introduced potential temporal and spatial sampling bias in our phylogeographic analyses.

It was hypothesized that the VOC Alpha may have arisen during a long-term persistent infection of SARS-CoV-2 as all mutations emerged simultaneously (Rambaut et al. 2020). The VOC Gamma, on the other hand, acquired its constellation of mutations through multiple interhost transmissions, which is revealed by our previous observations when describing the sub-lineage Gamma-like-I (Naveca et al. 2021) and is now confirmed by the identification of Gamma-like-II. During this stepwise evolutionary process that probably took several months, acquisition of mutations was not uniformly distributed along the viral genome. Most mutations located in the NTD (L18F, P26S, and D138Y) and in the RBD (K417T, E484K, and N501Y) of the S protein were fixed in the first evolutionary step, while most mutations located outside the S gene were fixed at subsequent steps. It is noteworthy that more genomes from diverse lineages representing additional intermediate evolutionary steps could exist between clade 28-AM-II and lineage P.1 ancestor, but the limited number of available genomes sampled in Amazonas between September and November 2020 (*n* = 87) limits the resolution of the evolutionary history reconstructed here.

A recent preprint study indicates that the emergence of VOCs was driven by an episodic increase in the evolutionary rate that was revealed when using the FLC-stem model, a clock model that assumes that VOC stem branches can have a rate that differs from the background (Tay et al. 2021). Our analyses indicate that both FLC-stem and relaxed clock models were similarly supported by our data. Both models provide similar tMRCA for Gamma and Gamma-like clades but somewhat different time-scales for the early diversification of lineage P.1. While the relaxed molecular clock model traced the origin of the P.1_MRCA1_ ancestor to early September 2020, the FLC-stem model pushed its origin to mid-October 2020. The shorter period of cryptic circulation of the lineage P.1 ancestor supported by the FLC-stem model is a more plausible epidemiological scenario considering the absence of RBD mutations among the SARS-CoV-2 sequences from the Amazonas state analyzed between September and November 2020 (Naveca et al. 2021).

Tay et al. estimated that the mean evolutionary rate for the P.1 + Gamma stem was 27.6 × 10^–4^ subs/site/year (Tay et al. 2021) while our estimates support that the median evolutionary rate ranges from 11.2 × 10^–4^ subs/site/year for the P.1_MRCA1_ stem to 49.1 × 10^–4^ subs/site/year for the Gamma stem, a 1.6- to 6.8-fold increase compared to the background rate (Table 2). Although the credibility interval of the stem rates was very broad and results should be interpreted with caution, our findings support that episodic acceleration of the evolutionary rate not only affected the basal P.1 stem but also extended to some internal P.1 branches and was thus not restricted to a single infection case. Furthermore, such episodic acceleration not only affected the fixation of mutations within the S protein but also drove mutations outside the S protein that occurred between the P.1_MRCA1_ and the P.1 sub-lineage ancestors. Elucidating the circumstances that accelerated viral mutations across multiple individuals during a limited time period will be crucial to understand the emergence of VOCs.

The stepwise diversification of lineage P.1 resembles the evolutionary pattern of the lineages B.1.351 and B.1.617 that were first detected in South Africa and India, respectively. Lineages B.1.351 and B.1.617 also comprise a family of related clades, including the VOCs Beta and Delta, respectively, with partial overlapping mutations. The mutation profile of lineage B.1.351 suggests that five non-synonymous mutations in the S protein (D80A, D215G, E484K, N501Y, and A701V) were fixed at the first progenitor and further S mutations (L18F, 242–244del, R246I, and K417N) were fixed at later steps in different descendant sub-lineages (Tegally et al. 2021). Lineage B.1.617 was initially defined as a double S mutant (L452R and E484), but subsequent phylogenetic analysis revealed a high within-lineage diversity with at least four different PANGO lineages, B.1.617, B.1.617.1, B.1.617.2 (VOC Delta), and B.1.617.3, which could be linked to partially overlapping constellations of S mutations (Cherian et al. 2021; Mlcochova et al. 2021).

Although this stepwise evolutionary pattern does not exclude the possibility that a subset of mutations, particularly those at the RBD of the S protein, could have originated in a long-term infected individual, sequential infections of such kind of patients are very unlikely. We propose that mutations in VOCs may have also been selected during acute reinfections of partially protected immunocompetent individuals. The partial immunity that human populations acquired through natural SARS-CoV-2 infections during early 2020 may have been a major selective force of mutations of concern in the second half of 2020. Remarkably, the high attack rates (30–76 per cent) estimated after the first epidemic wave in Manaus (Buss et al. 2021; He et al. 2021) combined with persistent viral circulation and weak mitigation measures (Naveca et al. 2021) may have created the optimal conditions for the occurrence of reinfections in the Amazonas by the late 2020. This model is also consistent with a recent study showing that the simultaneous expansion of different VOCs coincided with a major global change in the selective environment within which SARS-CoV-2 evolved since October 2020 (Martin et al. 2021).

Although key mutations of concern in the RBD of S protein (K417T, E484K, and N501Y) likely substantially increase viral transmissibility (Cai et al. 2021; Gobeil et al. 2021), some pieces of evidence suggest that these RBD mutations were not the only driver of the successful expansion of the VOC Gamma. First, our evolutionary reconstruction indicates that P.1 ancestors that harbor the three RBD mutations of concern circulated cryptically in the Amazonas state since September–October 2020 without causing a large outbreak. Second, although all P.1 sub-lineages harbor the same RBD mutations of concern and emerged at around the same time, the VOC Gamma reached a much higher overall prevalence (69 per cent) in Brazil by January–March 2021 than clades Gamma-like-I and II (<2 per cent). One hypothesis is that substitutions outside the S protein may have played key roles in infectivity modulation (Obermeyer et al. 2021). Another hypothesis is that viral mutations combined with human factors, such as the lack of social distancing measures and mass gatherings/super-spreading events (Gómez-Carballa et al. 2021), may have also contributed to the remarkable dissemination of the VOC Gamma in the Amazonas state and throughout Brazil afterward.

The time lag between the emergence of variant progenitors carrying key mutations of concern and the start of the epidemic wave in the Amazonas was also observed in South Africa and India. The emergence of the B.1.351 progenitor, which harbors key RBD mutations (K417N, E484K, and N501Y), was traced in South Africa around late August 2020, while the country’s COVID-19 epidemic wave associated with the VOC Beta only began at the end of October 2020 (Tegally et al. 2021). Similarly, the B.1.617 progenitor with key RBD mutations (E484Q and L452R) probably dates back before October 2020, while the COVID-19 epidemic wave associated with the VOC Delta only began in February 2021 (GISAID 2021; World Health Organization 2021). The divergent epidemic trajectories of the VOC Delta (B.1.167.2) that is spreading through the world with respect to sub-lineages B.1.167.1 (that dominates in India) and B.1.167.3 (that remained uncommon in India and elsewhere) (Elbe and Buckland-Merrett 2017; Mlcochova et al. 2021) also support a complex interplay between the presence of mutations of concern and the epidemic dynamics of SARS-CoV-2 lineages.

In summary, our findings reveal that VOC Gamma is part of a family of P.1 sub-lineages that evolved from a common ancestor that carried key mutations of concern in the RBD. These findings confirm that the entire constellation of mutations that define the VOC Gamma was acquired in a stepwise process during multiple interhost transmissions that might have been featured by episodic mutation rate acceleration. It further supports that multiple P.1 sub-lineages with the same RBD mutations co-circulated in the Amazonas months before the abrupt resurgence of COVID-19 in the state in late 2020 (Faria et al. 2021; Naveca et al. 2021). The period of cryptic circulation of lineage P.1 in Amazonas and the very divergent epidemic trajectories of P.1 sub-lineages suggest that mutations outside the RBD and/or non-virological (human behavior) factors probably drove the remarkable successful spread of the VOC Gamma in Brazil.

## Supplementary Material

veab091_Supp

## Data Availability

SARS-CoV-2 genome sequences generated in this study have been deposited in the GISAID platform (https://www.gisaid.org/), accession number IDs EPI_ISL_2038926 to EPI_ISL_2038968, EPI_ISL_2102018, EPI_ISL_2102063, EPI_ISL_2157408, EPI_ISL_2157421, EPI_ISL_2157485, EPI_ISL_2157488, EPI_ISL_2196259, EPI_ISL_2274118, EPI_ISL_2274121, EPI_ISL_2274122, EPI_ISL_2758945, EPI_ISL_2758953, EPI_ISL_2758957, EPI_ISL_2758958, EPI_ISL_2758962, EPI_ISL_2758964, EPI_ISL_2758966, EPI_ISL_2758972, and EPI_ISL_2775395.
