# Supplementary material for: Identification of a novel SARS-CoV-2 P.1 sub-lineage in Brazil provides new insights about the mechanisms of emergence of variants of concern
Source: Virus Evol. 2021 Dec 15;7(2):veab091. doi: 10.1093/ve/veab091 (PMC8754780; doi:10.1093/ve/veab091)
Supplement: veab091_Supp [file veab091_supp.zip › FigureS1.pdf]

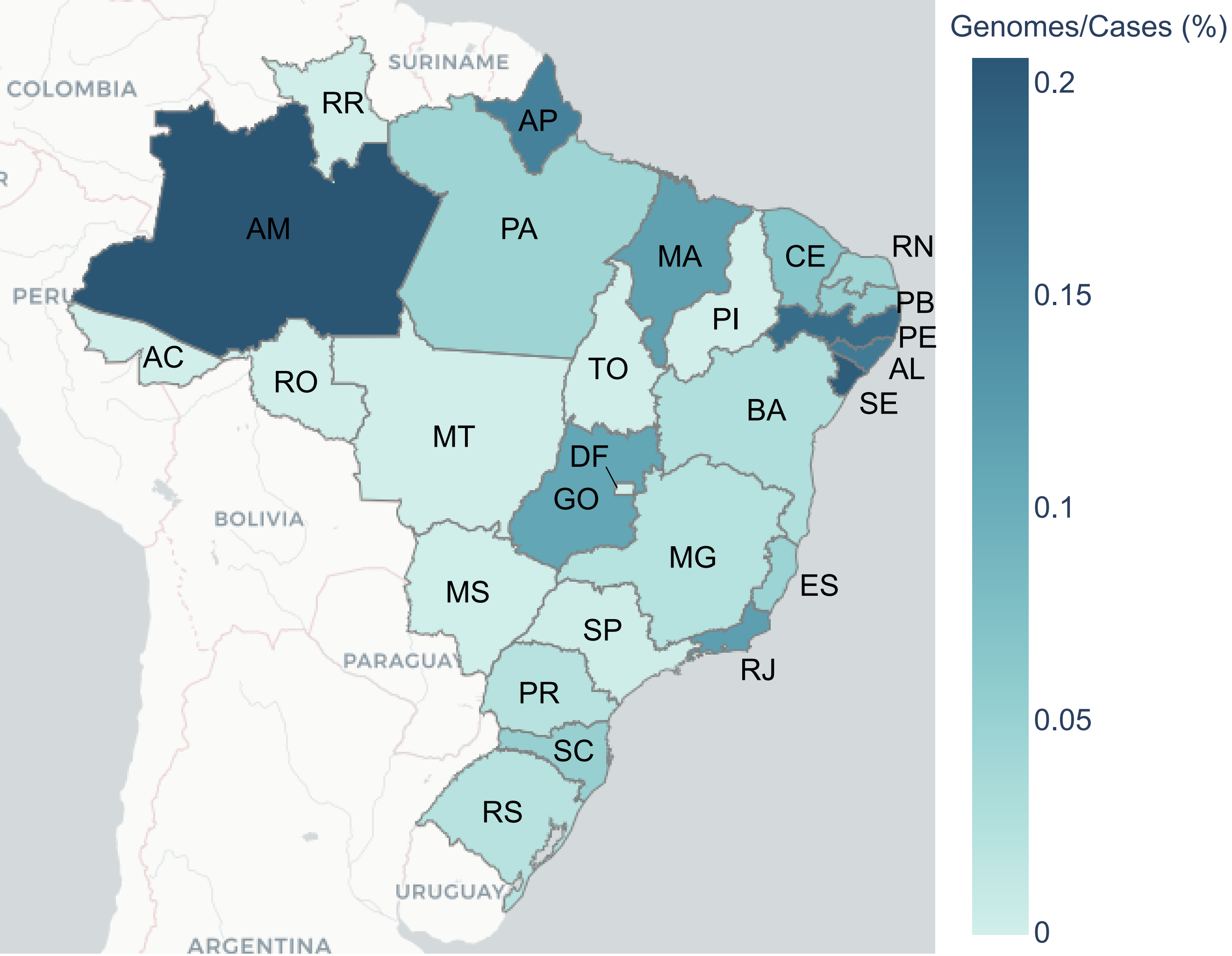

Figure S1 – Percentage of confirmed cases sequenced by the Fiocruz COVID-19 Genomic Surveillance Network in all Brazilian states between 1st August 2020 and 31st March 2021. Brazilian states' names follow the ISO 3166-2 standard.
