# Supplementary material for: Identification of a novel SARS-CoV-2 P.1 sub-lineage in Brazil provides new insights about the mechanisms of emergence of variants of concern
Source: Virus Evol. 2021 Dec 15;7(2):veab091. doi: 10.1093/ve/veab091 (PMC8754780; doi:10.1093/ve/veab091)
Supplement: veab091_Supp [file veab091_supp.zip › TableS1_Table_61genomes_P1-like-II.docx]

**Supplementary Table 1.** Epidemiological information of the SARS-CoV-2 Gamma-like-II clade GR genomes recovered in this study by COVID-19 Fiocruz Genomic Surveillance Network.

| **Virus name** | **Accession ID** | **Collection date** | **Location** | **Gender** | **Patient age** | **Patient status** | **Clinical Specimen** |
| --- | --- | --- | --- | --- | --- | --- | --- |
| hCoV-19/Brazil/SC-FIOCRUZ-9767/2021 | EPI_ISL_2038926 | 16/01/2021 | Santa Catarina | Male | unknown | unknown | NPS |
| hCoV-19/Brazil/SC-FIOCRUZ-9766/2021 | EPI_ISL_2038927 | 14/02/2021 | Santa Catarina | Female | unknown | unknown | NPS |
| hCoV-19/Brazil/SC-FIOCRUZ-9731/2021 | EPI_ISL_2038928 | 15/02/2021 | Santa Catarina / Itapema | unknown | 56 | unknown | NPS |
| hCoV-19/Brazil/SC-FIOCRUZ-9769/2021 | EPI_ISL_2038929 | 18/02/2021 | Santa Catarina | Female | unknown | unknown | NPS |
| hCoV-19/Brazil/SC-FIOCRUZ-11188/2021 | EPI_ISL_2038930 | 22/01/2021 | Santa Catarina / Chapeco | Female | 70 | Deceased | NPS |
| hCoV-19/Brazil/SC-FIOCRUZ-14559/2021 | EPI_ISL_2038931 | 03/02/2021 | Santa Catarina / Chapeco | Male | 62 | Deceased | NPS |
| hCoV-19/Brazil/SC-FIOCRUZ-14557/2021 | EPI_ISL_2038932 | 29/01/2021 | Santa Catarina / Chapeco | Female | 60 | Deceased | NPS |
| hCoV-19/Brazil/SC-FIOCRUZ-7173/2021^A^ | EPI_ISL_2038933 | 29/01/2021 | Santa Catarina / Chapeco | Female | 31 | unknown | NPS |
| hCoV-19/Brazil/SC-FIOCRUZ-14558/2021 | EPI_ISL_2038934 | 02/02/2021 | Santa Catarina / Chapeco | Male | 55 | Deceased | NPS |
| hCoV-19/Brazil/SC-FIOCRUZ-14560/2021 | EPI_ISL_2038935 | 05/02/2021 | Santa Catarina / Chapeco | Female | 67 | Deceased | NPS |
| hCoV-19/Brazil/SC-FIOCRUZ-14561/2021 | EPI_ISL_2038936 | 07/02/2021 | Santa Catarina / Chapeco | Female | 69 | Deceased | NPS |
| hCoV-19/Brazil/SC-FIOCRUZ-14562/2021 | EPI_ISL_2038937 | 07/02/2021 | Santa Catarina / Chapeco | Female | 63 | Deceased | NPS |
| hCoV-19/Brazil/SC-FIOCRUZ-14564/2021 | EPI_ISL_2038938 | 08/02/2021 | Santa Catarina / Chapeco | Male | 48 | Deceased | NPS |
| hCoV-19/Brazil/SC-FIOCRUZ-14565/2021 | EPI_ISL_2038939 | 08/02/2021 | Santa Catarina / Chapeco | Male | 46 | Deceased | NPS |
| hCoV-19/Brazil/SC-FIOCRUZ-14566/2021 | EPI_ISL_2038940 | 08/02/2021 | Santa Catarina / Chapeco | Female | 50 | Deceased | NPS |
| hCoV-19/Brazil/SC-FIOCRUZ-11193/2021 | EPI_ISL_2038941 | 09/02/2021 | Santa Catarina / Chapeco | Male | 77 | Deceased | NPS |
| hCoV-19/Brazil/SC-FIOCRUZ-14568/2021 | EPI_ISL_2038942 | 09/02/2021 | Santa Catarina / Chapeco | Male | 44 | Deceased | NPS |
| hCoV-19/Brazil/SC-FIOCRUZ-14569/2021 | EPI_ISL_2038943 | 09/02/2021 | Santa Catarina / Chapeco | Male | 68 | Deceased | NPS |
| hCoV-19/Brazil/SC-FIOCRUZ-14570/2021 | EPI_ISL_2038945 | 11/02/2021 | Santa Catarina / Chapeco | Female | 60 | Deceased | NPS |
| hCoV-19/Brazil/SC-FIOCRUZ-11189/2021 | EPI_ISL_2038946 | 16/02/2021 | Santa Catarina / Chapeco | Male | 90 | Deceased | NPS |
| hCoV-19/Brazil/SC-FIOCRUZ-11183/2021 | EPI_ISL_2038947 | 15/02/2021 | Santa Catarina / Sao Joaquim | Female | 31 | unknown | NPS |
| hCoV-19/Brazil/SC-FIOCRUZ-10872/2021 | EPI_ISL_2038948 | 21/02/2021 | Santa Catarina / Mafra | Female | 22 | unknown | NPS |
| hCoV-19/Brazil/SC-FIOCRUZ-10866/2021 | EPI_ISL_2038949 | 19/02/2021 | Santa Catarina / Braco do Norte | Female | 23 | unknown | NPS |
| hCoV-19/Brazil/SC-FIOCRUZ-11049/2021 | EPI_ISL_2038950 | 27/01/2021 | Santa Catarina / Chapeco | Female | 54 | unknown | NPS |
| hCoV-19/Brazil/SC-FIOCRUZ-11050/2021 | EPI_ISL_2038951 | 30/01/2021 | Santa Catarina / Chapeco | Male | 36 | unknown | NPS |
| hCoV-19/Brazil/SC-FIOCRUZ-11051/2021 | EPI_ISL_2038952 | 01/02/2021 | Santa Catarina / Chapeco | Male | 39 | unknown | NPS |
| hCoV-19/Brazil/SC-FIOCRUZ-11052/2021 | EPI_ISL_2038953 | 02/02/2021 | Santa Catarina / Chapeco | Female | 56 | unknown | NPS |
| hCoV-19/Brazil/SC-FIOCRUZ-14567/2021 | EPI_ISL_2038954 | 09/02/2021 | Santa Catarina / Chapeco | Male | 42 | Deceased | NPS |
| hCoV-19/Brazil/PR-FIOCRUZ-9305/2021 | EPI_ISL_2038955 | 12/02/2021 | Parana / Cascavel | Male | 59 | Hospitalized | NPS |
| hCoV-19/Brazil/RJ-FIOCRUZ-13895/2021 | EPI_ISL_2038956 | 10/03/2021 | Rio de Janeiro / Paraiba do Sul | Female | 42 | unknown | NPS |
| hCoV-19/Brazil/RS-FIOCRUZ-7363/2021 | EPI_ISL_2038957 | 27/01/2021 | Rio Grande do Sul / Gramado | Female | 53 | Hospitalized | NPS |
| hCoV-19/Brazil/RS-FIOCRUZ-7398/2021 | EPI_ISL_2038958 | 09/02/2021 | Rio Grande do Sul / Nonoai | Male | 56 | Hospitalized | NPS |
| hCoV-19/Brazil/RS-FIOCRUZ-14225/2021 | EPI_ISL_2038959 | 03/03/2021 | Rio Grande do Sul / Seberi | Female | 67 | unknown | NPS |
| hCoV-19/Brazil/RS-FIOCRUZ-14248/2021 | EPI_ISL_2038960 | 17/03/2021 | Rio Grande do Sul / Carazinho | Female | 68 | unknown | NPS |
| hCoV-19/Brazil/RJ-FIOCRUZ-6759/2021 | EPI_ISL_2038961 | 15/02/2021 | Rio de Janeiro / Rio de Janeiro | Female | 41 | unknown | NPS |
| hCoV-19/Brazil/RJ-FIOCRUZ-6760/2021 | EPI_ISL_2038962 | 15/02/2021 | Rio de Janeiro / Rio de Janeiro | Female | 39 | unknown | NPS |
| hCoV-19/Brazil/RJ-FIOCRUZ-7745/2021 | EPI_ISL_2038963 | 24/02/2021 | Rio de Janeiro / Rio de Janeiro | Male | 75 | unknown | NPS |
| hCoV-19/Brazil/AL-FIOCRUZ-8935/2021 | EPI_ISL_2038964 | 11/02/2021 | Alagoas / Maceio | Female | 21 | unknown | NPS |
| hCoV-19/Brazil/MG-FIOCRUZ-9385/2021 | EPI_ISL_2038965 | 22/02/2021 | Minas Gerais / Santo Antonio do Aventureiro | unknown | 25 | unknown | NPS |
| hCoV-19/Brazil/SC-FIOCRUZ-7174/2021^A^ | EPI_ISL_2038966 | 29/01/2021 | Santa Catarina / Florianopolis | Female | 52 | unknown | NPS |
| hCoV-19/Brazil/SC-FIOCRUZ-11054/2021 | EPI_ISL_2038967 | 28/02/2021 | Santa Catarina / Chapeco | Male | 59 | unknown | NPS |
| hCoV-19/Brazil/ES-FIOCRUZ-16386/2021^B^ | EPI_ISL_2038968 | 04/03/2021 | Espirito Santo / Serra | Male | 67 | unknown | NPS |
| hCoV-19/Brazil/AM-FIOCRUZ-21890078NR/2021 | EPI_ISL_2102018 | 22/03/2021 | Amazonas / Manaus | Male | 55 | unknown | NPS |
| hCoV-19/Brazil/AM-FIOCRUZ-21895614MA/2021 | EPI_ISL_2102063 | 25/03/2021 | Amazonas / Manaus | Female | 41 | unknown | NPS |
| hCoV-19/Brazil/PR-FIOCRUZ-19056/2021 | EPI_ISL_2157408 | 03/03/2021 | Parana / Toledo | unknown | 66 | Hospitalized | NPS |
| hCoV-19/Brazil/PR-FIOCRUZ-19062/2021 | EPI_ISL_2157421 | 07/03/2021 | Parana / Toledo | unknown | 76 | unknown | NPS |
| hCoV-19/Brazil/PR-FIOCRUZ-19075/2021 | EPI_ISL_2157485 | 15/03/2021 | Parana / Capitao Leonidas Marques | unknown | 74 | Deceased | NPS |
| hCoV-19/Brazil/PR-FIOCRUZ-19076/2021 | EPI_ISL_2157488 | 15/03/2021 | Parana / Toledo | unknown | 65 | Hospitalized | NPS |
| hCoV-19/Brazil/PR-FIOCRUZ-21060/2021 | EPI_ISL_2196259 | 23/03/2021 | Parana / Francisco Beltrao | Female | 45 | Hospitalized | NPS |
| hCoV-19/Brazil/RS-FIOCRUZ-21517/2021 | EPI_ISL_2274118 | 31/03/2021 | Rio Grande do Sul / Ijui | Male | 19 | unknown | NPS |
| hCoV-19/Brazil/RS-FIOCRUZ-21505/2021 | EPI_ISL_2274121 | 16/03/2021 | Rio Grande do Sul / Nonoai | Female | 51 | unknown | NPS |
| hCoV-19/Brazil/RS-FIOCRUZ-21502/2021 | EPI_ISL_2274122 | 04/03/2021 | Rio Grande do Sul / Nonoai | Male | 22 | unknown | NPS |
| hCoV-19/Brazil/PR-FIOCRUZ-HF109/2021 | EPI_ISL_2758945 | 02/03/2021 | Parana | unknown | unknown | unknown | NPS |
| hCoV-19/Brazil/PR-FIOCRUZ-HF117/2021 | EPI_ISL_2758953 | 06/03/2021 | Parana | unknown | unknown | unknown | NPS |
| hCoV-19/Brazil/PR-FIOCRUZ-HF122/2021 | EPI_ISL_2758957 | 03/03/2021 | Parana | unknown | unknown | unknown | NPS |
| hCoV-19/Brazil/PR-FIOCRUZ-HF124/2021 | EPI_ISL_2758958 | 05/03/2021 | Parana | unknown | unknown | unknown | NPS |
| hCoV-19/Brazil/PR-FIOCRUZ-HF130/2021 | EPI_ISL_2758962 | 02/03/2021 | Parana | unknown | unknown | unknown | NPS |
| hCoV-19/Brazil/PR-FIOCRUZ-HF132/2021 | EPI_ISL_2758964 | 01/03/2021 | Parana | unknown | unknown | unknown | NPS |
| hCoV-19/Brazil/PR-FIOCRUZ-HF135/2021 | EPI_ISL_2758966 | 07/03/2021 | Parana | unknown | unknown | unknown | NPS |
| hCoV-19/Brazil/PR-FIOCRUZ-HF142/2021 | EPI_ISL_2758972 | 04/03/2021 | Parana | unknown | unknown | unknown | NPS |
| hCoV-19/Brazil/PR-FIOCRUZ-HF134/2021 | EPI_ISL_2775395 | 06/03/2021 | Parana | unknown | unknown | unknown | NPS |

1. Healthcare worker in direct contact with patient positive for SARS-CoV-2 from Manaus, Amazonas
2. Patient from Chapeco, Santa Catarina transferred to a hospital in Serra, Espirito Santo

NPS = nasopharyngeal swab and NPA = nasopharyngeal aspirate
