# Supplementary material for: Identification of a novel SARS-CoV-2 P.1 sub-lineage in Brazil provides new insights about the mechanisms of emergence of variants of concern
Source: Virus Evol. 2021 Dec 15;7(2):veab091. doi: 10.1093/ve/veab091 (PMC8754780; doi:10.1093/ve/veab091)
Supplement: veab091_Supp [file veab091_supp.zip › TableS2 - GSS.docx]

**Table S2** – Bayes Factor support estimated with the generalized stepping stone sampling (GSS) comparing (column X row) different clock models.

| Clock Model | Strict | Relaxed UCLD | RLC | FLC-stem |
| --- | --- | --- | --- | --- |
| Strict | - | 3.1 | <100 | 1.4 |
| Relaxed UCLD | - | - | <100 | -1.7 |
| RLC | - | - | - | >100 |
| FLC-stem | - | - | - | - |

UCLD – Uncorrelated Log-normal Distribution; RLC – Random Local Clock; FLC – Fixed Local Clock
